# Supplementary material for: A solution for constraining past marine Polar Amplification
Source: Nat Commun. 2024 Oct 18;15:9002. doi: 10.1038/s41467-024-53424-w (PMC11489591; doi:10.1038/s41467-024-53424-w)
Supplement: Supplementary file 1 — Supplementary Information [file 41467_2024_53424_MOESM1_ESM.pdf]

Supplementary Materials for:

A solution for constraining past marine Polar Amplification

Morley, A.[1,2*], de la Vega, E.[1], Raitzsch, M.[3], Bijma, J.[4], Ninnemann, U.[5], Foster, G.L.[6], Chalk, T.B.[7], Meilland, J.[8], Cave, R.R.[9], Büscher, J. V.[9,10], and Kucera, M[8].

[1] *University of Galway, School of Geography, Archaeology and Irish Studies, H91TK33 Galway, Ireland.*
[2] *iCRAG – Irish Centre for Research in Applied Geosciences, Belfield, Dublin 4, Ireland.*
[3] *Dettmer Group GmbH & Co. KG.,* 28195 *Bremen, Germany*
[4] *Alfred-Wegener-Institut, Helmholtz-Zentrum für Polar- und Meeresforschung, Am Handelshafen 12, 27570, Bremerhaven, Germany*
[5] *University of Bergen, Department of Earth Science and Bjerknes Centre for Climate Research, 5007, Bergen, Norway*
[6] *School of Ocean and Earth Science, University of Southampton, National Oceanography Centre Southampton, Southampton, SO14 3ZH, UK*
[7] *Centre Européen de Recherche et d'enseignement des géosciences de l'environnement (CEREGE), Aix-en-Provence, France*
[8] *MARUM – Center for Marine Environmental Sciences, University of Bremen, Bremen, Germany*
[9] *University of Galway, School of Natural Sciences, H91TK33 Galway, Ireland*
[10] *Ulster University, School of Geography and Environmental Sciences, BT52 1SA, Coleraine, UK*
* *Email: audrey.morley@universityofgalway.ie*

Content

Supplementary Figures

- **Supplementary Figure S1** Station Map of all samples used in this study.
- **Supplementary Figure S2** Regression Tree describing the interaction of $[CO_3^{2-}]$ and temperature on $\delta^{18}O_c$.
- **Supplementary Figure S3** shows the relationship between Temperature and $\delta^{18}O_c$ and $[CO_3^{2-}]$
- **Supplementary Figure S4** shows the regression and residuals for the $\delta^{18}O_c$ and $[CO_3^{2-}]$ relationship.
- **Supplementary Figure S5** shows the uncorrected LGM and Late Holocene SSTs based on *N. pachyderma* Mg/Ca.

Supplementary Tables

- **Supplementary Table 1** Hydrographic datasets used for each station of this study.
- **Supplementary Table 2** Hydrographic data and Oxygen Isotopes measured on living *N. pachyderma* from plankton tows.
- **Supplementary Table 3** Hydrographic data and Mg/Ca measured on living *N. pachyderma* from plankton tows.
- **Supplementary Table 4** Hydrographic data and Mg/Ca with Oxygen Isotopes measured on dead *N. pachyderma* from plankton tows and core tops.
- **Supplementary Table 5** Generalised Linear Model Results.
- **Supplementary Table 6** ΔSST calculations using MAT-derived SST, uncorrected and corrected Mg/Ca values measured south of Greenland (Eirik Drift) on *N. pachyderma.*
- **Supplementary Table 7** Materials and time intervals used to calculate ΔSST for the Holocene-LGM
- **Supplementary Table 8** Hydrographic data, stable isotopes and Mg/Ca used for the reanalysis of Kozdon et al. [1]

Supplementary Figures

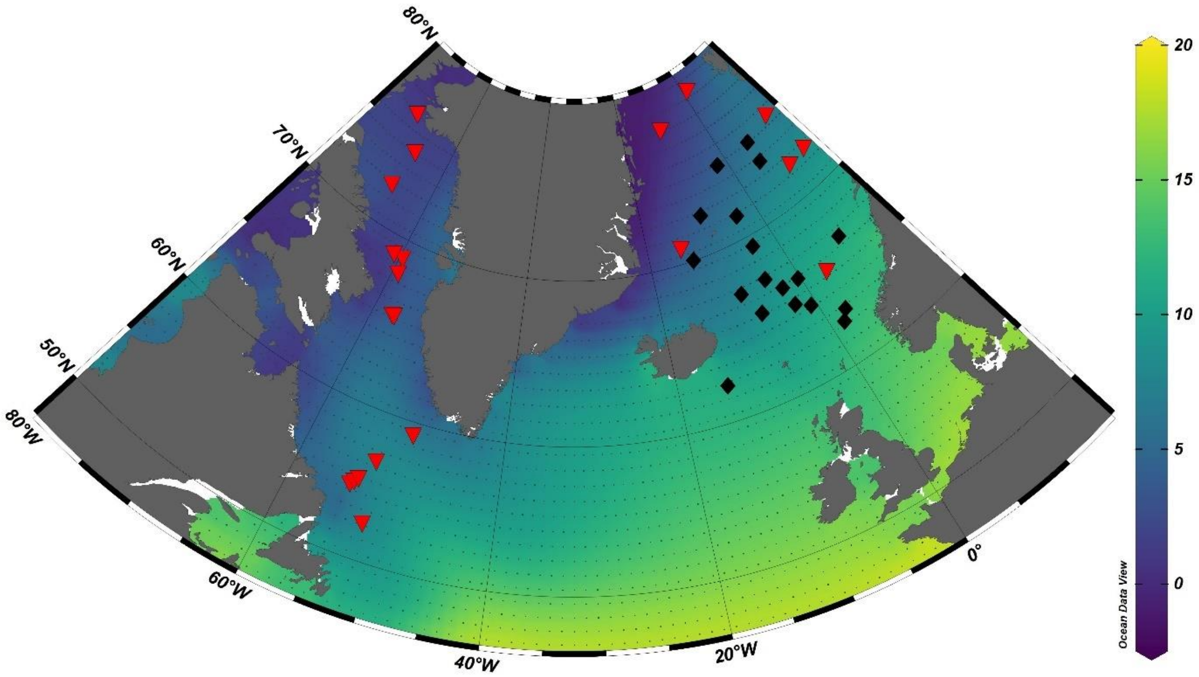

Supplementary Figure S1 Station Map. Inverted triangles (red) show samples collected from plankton tows (e.g., MSM cruises in the Baffin Bay and Labrador Sea and CE cruises in the Nordic Sea), while black triangles show locations of the Kozdon dataset. In the Nordic Seas plankton tows are also paired with corresponding core tops. The temperature data shown is from the June-September WOA 2018 dataset at 30m [2]. The map was generated using Ocean Data View, [3].

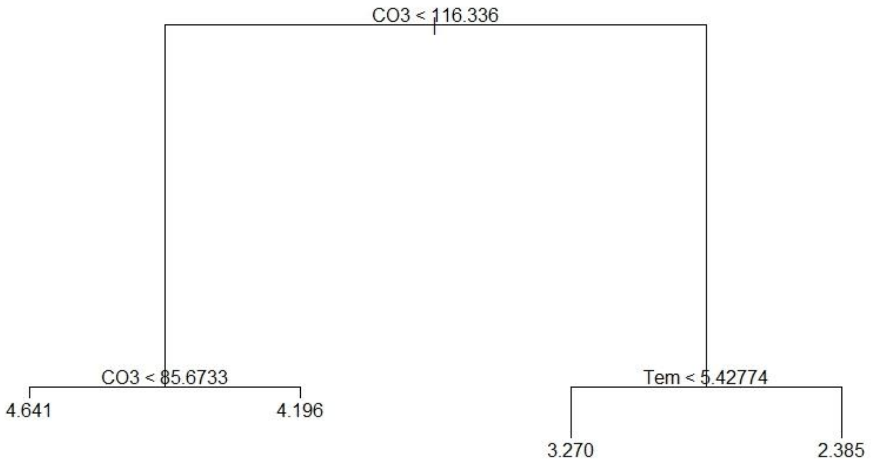

Supplementary Figure S2 Regression Tree. This plot describes the interaction of $[CO_3^{2-}]$ and temperature on $\delta^{18}O_c$ in the generalized model.

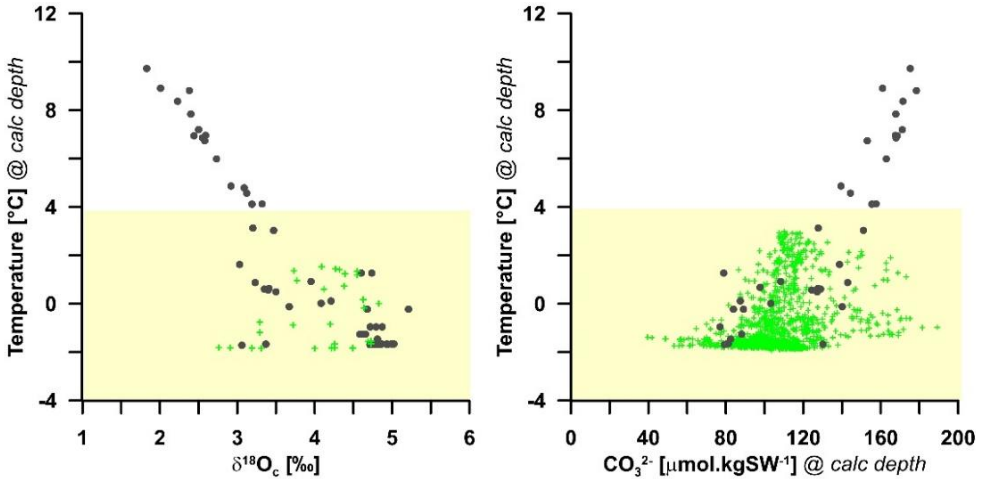

Supplementary Figure S3. The left panel shows the relationship between Temperature and $\delta^{18}O_c$, while the right panel shows the relationship between Temperature and $[CO_3^{2-}]$ for the combined tow and core top *N. pachyderma* datasets. The plankton tow dataset on *N. pachyderma* (left) and associated hydrographic data (derived using GlODAP (right) from Bauch et al. [4] is shown in green. The yellow shading highlights hydrographic conditions below 4°C.

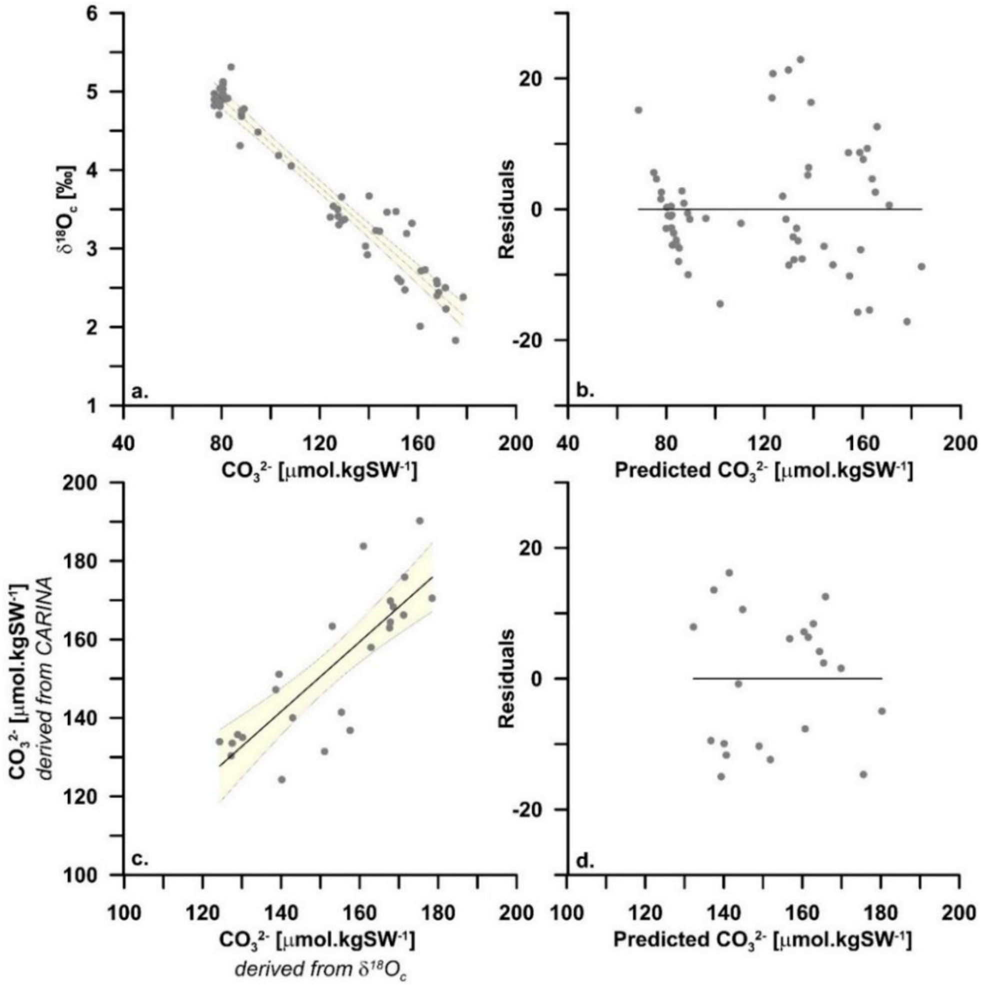

Supplementary Figure S4 Residual Plots. (Top) shows the regression and residuals for the $\delta^{18}O_c$ and $[CO_3^{2-}]$ relationship. (Bottom) shows the regression and residuals for the relationship describing $[CO_3^{2-}]$ derived from CARINA and $[CO_3^{2-}]$ derived from $\delta^{18}O_c$.

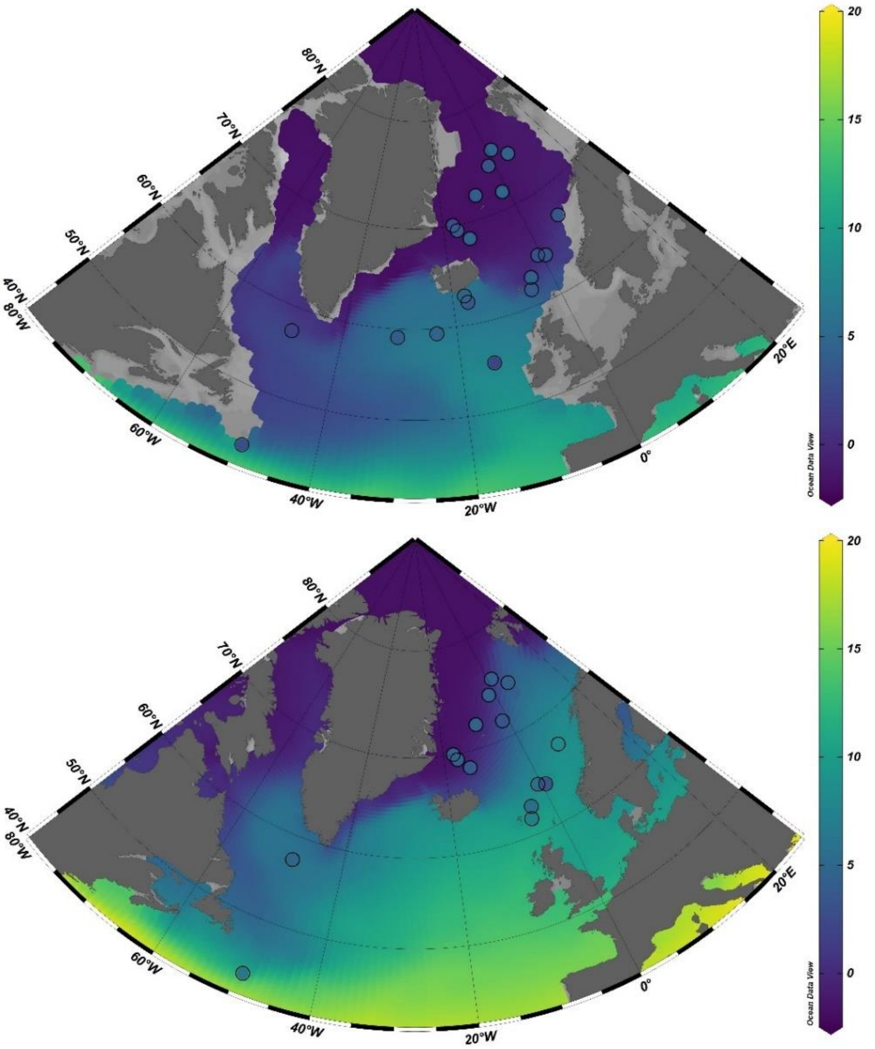

Supplementary Figure S5 Uncorrected LGM (top) and Late Holocene (bottom) sea surface temperature (SSTs) based on *N. pachyderma* Mg/Ca calibrated using the calibration equation developed by Kozdon et al. 2009 [1]. For datasets used please see Supplementary Table 7. Late Holocene and the Last Glacial Maximum SSTS were calculated using the isotope-enabled Community Earth System Model (iCESM) as shown in Tierney et al.[5]. The map was generated using Ocean Data View, [3].

Supplementary References:

1. Kozdon, R., et al., *Reassessing Mg/Ca temperature calibrations of Neogloboquadrina pachyderma (sinistral) using paired δ44/40Ca and Mg/Ca measurements.* Geochemistry, Geophysics, Geosystems, 2009. **10**(3).
2. Locarnini, M., et al., *World ocean atlas 2018, volume 1: Temperature.* 2018.
3. Schlitzer, R., *Ocean data view.* 2022.
4. Bauch, D., J. Carstens, and G. Wefer, *Oxygen isotope composition of living Neogloboquadrina pachyderma (sin.) in the Arctic Ocean.* Earth and Planetary Science Letters, 1997. **146**(1-2): p. 47-58.
5. Tierney, J.E., et al., *Glacial cooling and climate sensitivity revisited.* Nature, 2020. **584**(7822): p. 569-573.

Supplementary Table 1. Hydrographic datasets used for station of this study.

Cruise	ID	Lat	Long	Date	Hydrographic data [survey, station ID, location, date, data]	offset tow -hydro
MSM09	GeoB455	68° 58.12' N	59° 34.38' W	04/09/2008	Davies Strait Time Series; Stn. 34 (68.4965N, 59.69W), 36 (68.539N, 59.338W), 37 (68.5695N, 59.098W) 08.09.2008 [T, S, DIC, Alk, Silicate, Phosphate, d18Osw]	4 days
MSM09	GeoB415	52° 40.01' N	51° 57.99' W	08/22/2008	CLIVAR_LINE_2008; Station 3; 22/08/2008; 53.6757N, 55.5365W [T, S, DIC, Alk, Silicate, Phosphate]	0 day
MSM09	GeoB433	54° 55.98' N	54° 20.09' W	26/08/2008	CLIVAR_LINE_2008; Station 6 and or 13; 22/08/2008; 53.6757N, 55.5365W [T, S, DIC, Alk, Silicate, Phosphate]	0 day
MSM09	GeoB434	55° 10.76' N	54° 02.30' W	26/08/2008	CLIVAR_LINE_2008; Closest Station 13; 26/08/2008; 55.9067N, 53.408W [T, S, DIC, Alk, Silicate, Phosphate]	0 day
MSM09	GeoB435	55° 26.98' N	53° 43.96' W	26/08/2008	CLIVAR_LINE_2008; Closest Station 13; 26/08/2008; 55.9067N, 53.408W [T, S, DIC, Alk, Silicate, Phosphate]	0 day
MSM09	GeoB439	56° 53.19' N	52° 41.38' W	30/08/2008	CLIVAR_LINE_2008; Closest Station 17; 26/08/2008; 56.5432N, 52.673W [T, S, DIC, Alk, Silicate, Phosphate]	-4 days
MSM09	GeoB448	59° 14.00' N	49° 57.53' W	01/09/2008	CLIVAR_LINE_2008; Closest Station 23; 2/08/2008; 59.0545N, 49.948W [T, S, DIC, Alk, Silicate, Phosphate]	-30 days
MSM09	GeoB460	72° 20.57' N	67° 21.41' W	07/09/2008	CTD used for Temperature, GLODAP for Carb Chem 11.08.1997 [T, S, DIC, Alk, Silicate, Phosphate]	-11 years
MSM44	GeoB19913-2	65° 42.29' N	57° 7.63' W	03/07/2015	ArcticNet/ Geotracers; Station 39-BB1, 66°51.502N, 59°04.450W, 03/08/2015 [T, S, DIC, Alk, Silicate, Phosphate] GLODAP for d18Osw	31 days
MSM44	GeoB19914-2	65° 42.66' N	57° 26.77' W	03/07/2015	ArcticNet/ Geotracers; Station 39-BB1, 66°51.502N, 59°04.450W, 03/08/2015 [T, S, DIC, Alk, Silicate, Phosphate]	31 days
MSM44	GeoB19929-3	74° 34.49' N	67° 13.10' W	10/07/2015	GLODAP Station 209 & 211 75.311N, 67.002W, 31/07/2003 - Profiles match CTD and pH sensor very well. [T, S, DIC, Alk, Silicate, Phosphate, d18Osw]	-12 years, 21 days
MSM66	GeoB22365-3	69° 0.003´N	61° 4.864´W	20/08/2017	CCGS Amundsen - 2019-July Stations BB15 (68 27.08N 55 53.99W) and 227 (70 47.81N 56 59.27W) [T, S, DIC, Alk, Silicate, Phosphate] GLODAP for d18Osw	2 years, 30 days
MSM66	GeoB22304-3	68° 4.179´N	59°8.640´W	24/07/2017	CCGS Amundsen - 2019-July Stations BB15 (68 27.08N 55 53.99W) and 227 (70 47.81N 56 59.27W) [T, S, DIC, Alk, Silicate, Phosphate]	2 years, 30 days
MSN66	GeoB22323-3	76° 23.18´N	71°49.651´W	03/08/2017	CCGS Amundsen - AMU201702, ROV Pond 01/08/2017 77.6037N, -77.6037E [T, S, DIC, Alk, Silicate, Phosphate] GLODAP for d18Osw	29 days
CE20009	CE20009_02	70° 55.27'N	14° 21.5' E	30/08/2020	this study [T, S, DIC, Alk, Silicate, Phosphate, d18Osw]	paired
CE20009	CE20009_05	77° 37.19'N	09° 56.8' E	02/09/2020	this study [T, S, DIC, Alk, Silicate, Phosphate, d18Osw]	paired
CE20009	CE20009_10	75° 00.00'N	11° 85.28' W	05/09/2020	this study [T, S, DIC, Alk, Silicate, Phosphate, d18Osw]	paired
CE20009	CE20009_11	73° 09.41'N	18° 04.48' W	06/09/2020	this study [T, S, DIC, Alk, Silicate, Phosphate, d18Osw]	paired
CE20009	CE20009_12	70° 29,57'N	17° 55,49' W	07/09/2020	this study [T, S, DIC, Alk, Silicate, Phosphate, d18Osw]	paired
CE20009	CE20009_16	65° 48,07'N	03° 29,35' W	10/09/2020	this study [T, S, DIC, Alk, Silicate, Phosphate, d18Osw]	paired

Supplementary Table 2. Hydrographic data and Oxygen Isotopes measured on living N. pachyderma from plankton tows. See also Fig. 1

Cruise	Site	Tow Depth interval [m]		Dead/ life	size	weight [µg]	Species	Lab. Ident	d18Oc VPDB [‰]	d18Oc SD [‰]	d18Oc VPDB size norm [‰]	Hydro data depth [m]	T [C]	SAL [psu]	d18Osw [‰]	CO3 [µmol/kgSW]
MSM09	455	20	40	live	200-250	21.84	N. pachy.	C_021231	2.08	0.01	2.08	30-40	0.01	32.59	-1.26	103.21
MSM09	455	60	80	live	200-250	41.70	N. pachy.	C_021241	2.66	0.00	2.66	60-80	-1.26	33.41	-0.94	88.10
MSM09	455	60	80	live	200-250	41.70	N. pachy.	C_021242	2.61	0.02	2.61	60-80	-1.26	33.41	-0.94	88.10
MSM09	455	60	80	live	200-250	41.70	N. pachy.	C_021243	2.58	0.01	2.58	60-80	-1.26	33.41	-0.94	88.10
MSM09	455	100	140	live	250	27.90	N. pachy.	C_021259	3.21	0.02	3.07	100*	-0.23	33.74	-0.64	83.87
MSM66	22304-3	20	50	live	250-200	43.52	N. pachy.	C_021266	1.20	0.02	1.20	20-40	3.13	33.51	-0.95	127.76
MSM66	22365-3	10	30	live	150-200	21.07	N. pachy.	C_021278	1.12	0.02	1.12	10-30	4.57	33.47	-0.99	144.41
MSM66	22365-3	30	60	live	100-150	30.60	N. pachy.	C_021283	1.95	0.02	2.09	30-60	0.92	33.60	-0.88	108.32
MSM66	22365-3	60	90	live	150	28.22	N. pachy.	C_021285	2.21	0.01	2.35	60-90	0.11	33.76	-0.75	87.48
MSM66	22365-3	120	150	live	200-250	43.20	N. pachy.	C_021294	2.74	0.04	2.74	120-150	1.27	34.03	-0.52	78.89
MSM66	22365-3	120	150	live	150	51.80	N. pachy.	C_021295	2.60	0.01	2.60	120-150	1.27	34.03	-0.52	79.68
MSM44	19913-2	40	60	live	250-300	37.07	N. pachy.	C_021314	2.78	0.03	2.64	40-60	-1.69	32.95	-0.99	79.36
MSM44	19913-2	40	60	live	250-300	37.07	N. pachy.	C_021315	2.94	0.02	2.80	40-60	-1.69	32.95	-0.99	79.36
MSM44	19913-2	40	60	live	200-250	39.42	N. pachy.	C_021316	2.81	0.02	2.81	40-60	-1.69	32.95	-0.99	79.36
MSM44	19913-2	40	60	live	200-250	39.42	N. pachy.	C_021318	2.75	0.01	2.75	40-60	-1.69	32.95	-0.99	79.36
MSM44	19913-2	40	60	live	150-200	50.60	N. pachy.	C_021319	2.71	0.02	2.71	40-60	-1.69	32.95	-0.99	79.36
MSM44	19913-2	40	60	live	150-200	50.60	N. pachy.	C_021320	2.78	0.02	2.78	40-60	-1.69	32.95	-0.99	79.36
MSM44	19913-2	60	80	live	150-200	18.00	N. pachy.	C_021322	2.85	0.02	2.85	60-80	-1.69	33.09	-0.91	79.81
MSM44	19913-2	80	100	live	250-300	42.72	N. pachy.	C_021325	3.02	0.02	2.88	80-100	-1.66	33.22	-0.84	80.58
MSM44	19913-2	80	100	live	250-300	42.72	N. pachy.	C_021326	2.99	0.02	2.85	80-100	-1.66	33.22	-0.84	80.58
MSM44	19913-2	80	100	live	200-250	37.23	N. pachy.	C_021327	2.93	0.03	2.93	80-100	-1.66	33.22	-0.84	80.58
MSM44	19913-2	80	100	live	150-200	41.20	N. pachy.	C_021328	2.86	0.02	2.86	80-100	-1.66	33.22	-0.84	80.58
MSM44	19913-2	80	100	live	150-200	38.11	N. pachy.	C_021329	2.82	0.02	2.82	80-100	-1.66	33.22	-0.84	80.58
MSM44	19913-2	10	100	live	250-300	29.70	N. pachy.	C_021332	2.81	0.04	2.67	20-100	-1.46	32.95	-0.99	82.46
MSM44	19913-2	100	200	live	200-250	43.50	N. pachy.	C_021340	2.81	0.02	2.81	100-140	-1.65	33.37	-0.75	81.33
MSM44	19929-3	20	40	live	250-300	48.04	N. pachy.	C_021380	2.68	0.01	2.54	30-40	-0.23	33.33	-1.13	89.18
MSM44	19929-3	100	200	live?	250-300	21.00	N. pachy.	C_021410	2.87	0.03	2.73	100*	-0.96	33.81	-0.88	77.05

Supplementary Table 2 cont.

Cruise	Site	Tow Depth interval [m]		Dead/ life	size	weight [µg]	Species	Lab. Ident	d18Oc VPDB [‰]	d18Oc SD [‰]	d18Oc VPDB size norm [‰]	Hydro data depth [m]	T [C]	SAL [psu]	d18Osw [‰]	CO3 [µmol/kgSW]
MSM44	19929-3	100	200	live?	150-200	35.15	N. pachy.	C_021412	2.72	0.02	2.72	100*	-0.96	33.81	-0.88	77.05
MSM44	19929-3	200	300	live?	150-200	35.00	N. pachy.	C_021416	2.80	0.02	2.80	100*	-0.96	33.81	-0.76	77.05
CE20009	02	*0*	*10*	live	250-300	2.94	N. incom	na	na	na	na	5	10.70	34.72	0.34	168.73
CE20009	02	10	20	live	250-300	3.15	N. incom	na	0.95	0.08	0.95	10	10.60	34.75	0.36	160.76
CE20009	02	20	30	live	250-300	2.87	N. incom	na	1.16	0.08	1.16	30	10.21	34.83	0.42	131.73
CE20009	05	0	40	live	250-300	1.85	N. incom	na	1.59	0.08	1.59	5-40	7.47	34.84	0.61	141.62
CE20009	10	0	50	live	250-300	0.81	N. pachy.	na	na	na	na	5-60	3.72	34.47	0.17	128.53
CE20009	10	40	100	live	250-300	1.43	N. pachy.	na	na	na	na	40-100	2.48	34.48	0.25	121.40
CE20009	11	40	100	live	250-300	1.14	N. pachy.	na	2.48	0.08	2.48	40-100	-1.41	33.00	-0.87	94.79

** For these deep nets with large depth intervals (e.g., 100-200m) hydrographic data at 100m was chosen as it is unlikely that much deeper depth woud be representative*

Supplementary Table 3. Hydrographic data and Mg/Ca measured on living N. pachyderma from plankton tows. See also Fig. 1

Cruise	Site	Tow Depth interval [m]		Dead/ life	size max Ø [um]	size SD [um]	weight per ind [µg]	Species	Hydro data depth [m]	T [C]	S [psu]	CO3 [µmol/ kgSW]	Mg/Ca [mmol/ mol]
MSM44	GeoB19913-2	0	20	live	214	43	1.40	N. pachy.	0-20	0.68	32.10	106.70	1.44
MSM44	GeoB19913-2	0	100	live	252	29	2.36	N. pachy.	0-100	0.08	32.48	99.98	1.20
MSM44	GeoB19913-2	100	200	live	249	28	2.13	N. pachy.	100-200	-1.16	33.51	79.58	1.22
MSM44	GeoB19929-3	60	80	live	231	42	2.49	N. pachy.	60-80	-1.34	33.66	86.98	1.82
MSM44	GeoB19929-3	80	100	live	272	31	3.52	N. pachy.	80-100	-1.13	33.76	78.14	1.98
MSM44	GeoB19929-3	100	200	live	na	na	3.25	N. pachy.	100-125	-0.70	33.94	72.20	1.30
MSM66	GeoB22365-3	90	120	live	203	37	1.63	N. pachy.	90-120	-1.65	33.44	87.93	1.91
MSM66	GeoB22365-3	120	150	live	202	21	1.82	N. pachy.	120-150	-1.42	33.56	85.80	1.05
MSM09	GeoB455	80	100	live	205	26	2.10	N. pachy.	80-100	-1.08	33.54	88.33	0.98
MSM09	GeoB415	20	40	live	245	23	1.67	N. pachy.	10-40	1.48	32.23	101.54	1.58
MSM09	GeoB415	40	60	live	202	44	1.61	N. pachy.	40-60	-0.73		91.20	1.87
MSM09	GeoB433	100	150	live	243	22	2.04	N. pachy.	100-150	4.02	34.70	100.64	1.47
MSM09	GeoB433	100	150	live	243	22	2.04	N. pachy.	100-150	4.02	34.70	100.64	1.29
MSM09	GeoB434	0	20	live	258	24	2.95	N. pachy.	10-20	8.78	33.31	118.81	1.61
MSM09	GeoB434	0	20	live	258	24	2.95	N. pachy.	10-20	8.78	33.31	118.81	1.58
MSM09	GeoB434	20	40	live	na	na	3.14	N. pachy.	20-40	4.75	33.79	122.90	1.62
MSM09	GeoB434	20	40	live	na	na	3.14	N. pachy.	20-40	4.75	33.79	122.90	1.63
MSM09	GeoB435	100	150	live	na	na	2.08	N. pachy.	125	4.13	34.70	100.69	2.75
MSM09	GeoB435	100	150	live	na	na	2.08	N. pachy.	125	4.13	34.70	100.69	2.05
MSM09	GeoB439	0	20	live	na	na	1.83	N. pachy.	10-20	11.57	34.15	152.22	1.87
MSM09	GeoB439	0	20	live	na	na	1.83	N. pachy.	10-20	11.57	34.15	152.22	2.02
MSM09	GeoB460	40	60	live	na	na	0.99	N. pachy.	40-60	-1.37	33.07	88.17	1.46
MSM44	GeoB19914-2	40	60	live	na	na	1.41	N. pachy.	40-60	-1.69	32.95	79.36	1.35
MSM44	GeoB19914-2	40	60	live	na	na	1.41	N. pachy.	40-60	-1.69	32.95	79.36	1.37
CE20009	*02_01_05*	*0*	*10*	live	na	na	2.94	N. incom	5	10.70	34.72	168.73	1.66
CE20009	02_01_04	10	20	live	na	na	3.15	N. incom	10	10.60	34.75	160.76	1.67
CE20009	02_01_03	20	30	live	na	na	2.87	N. incom	30	10.21	34.83	131.73	1.81
CE20009	05_01_05	0	40	live	na	na	1.85	N. incom	5-40	7.47	34.84	141.62	1.43
CE20009	10_01_05	0	50	live	na	na	0.81	N. pachy.	5-60	3.72	34.47	128.53	1.67
CE20009	10_01_04	40	100	live	na	na	1.43	N. pachy.	40-100	2.48	34.48	121.40	1.39
CE20009	11_01_04	40	100	live	na	na	1.14	N. pachy.	40-100	-1.41	33.00	94.79	1.33

Supplementary Table 4. Hydrographic data and Mg/Ca with Oxygen Isotopes measured on dead N. pachyderma from plankton tows and core tops. See also Fig 1

Cruise	Site	Tow Depth interval [m]		Dead/ life	size [um]	weight /ind [µg]	Species	Hydro data depth [m]	T [C]	S [psu]	Mg/Ca [mmol/ mol]	CO3 [µmol/ kgSW]	Mg/Ca NORM	d18Oc VPDB
MSM09	GeoB455	80	100	dead	na	2.88	N. pachy.	80-100	-1.08	33.54	1.23	85.27	3.39	2.86
MSM09	GeoB455	100	140	dead	na	2.76	N. pachy.	100-140	-1.04	33.74	2.10	73.00	5.77	3.32
MSM44	GeoB19929-2	100	200	dead	na	5.34	N. pachy.	20	3.74	33.62	1.59	124.86	2.83	2.87
MSM44	GeoB19929-2	100	200	dead	na	5.34	N. pachy.	20	3.74	33.62	1.57	124.86	2.81	2.91
MSM44	GeoB19929-3	80	100	dead	na	5.52	N. pachy.	20	3.74	33.42	1.64	124.86	2.92	2.88
MSM44	GeoB19929-3	80	100	dead	na	5.52	N. pachy.	20	3.74	33.42	1.24	124.86	2.21	2.25
CE20009	CE20009_02_03	2170		core tops	200-250	na	N. pachy.	85	6.69	35.31	1.11	154.70	1.48	2.47
CE20009	CE20009_05_03	1296		core tops	200-250	na	N. pachy.	47	6.36	35.07	1.16	161.33	1.63	2.72
CE20009	CE20009_09_03	1985		core tops	200-250	na	N. pachy.	91	2.32	34.80	0.81	125.57	1.64	3.54
CE20009	CE20009_10_03	2637		core tops	200-250	na	N. pachy.	50	2.64	34.58	1.00	128.89	1.98	3.66
CE20009	CE20009_12_03	1674		core tops	200-250	na	N. pachy.	33	1.10	34.01	0.94	147.42	2.12	3.46
CE20009	CE20009_16_03	2890		core tops	200-250	na	N. pachy.	64	5.30	34.89	0.99	151.84	1.54	2.62

Supplementary Table 5. Generalised Linear Model Results

GLM $\delta^{18}O_c$	Factor	p val	signif. Code	Residual deviance	AIC
model 1 (T*dw)	T	7.16E-11	***	5.7301	42.883
	dw	0.0827	.		
interaction	**T:dw**	**0.035**	*		
model 2 (CO3*dw)	dw	0.823		7.2148	2.8858
	CO3	1.79E-14	***		
interaction	**CO3:dw**	**0.829**			
model 3 (T*CO3)	T	0.0171	*	2.256	-5.5875
	CO3	9.07E-12	***		
interaction	**T:CO3**	**0.1442**			

Signif. codes: 0 '***' 0.001 '**' 0.01 '*' 0.05 '.' 0.1 ' ' 1

Supplementary Table 6. ΔSST calculations using MAT derived SST, uncorrected and corrected Mg/Ca values measured south of Greenland (Eirik Drift) on N. pachyderma [32, 33, 58] See also Fig. 2

**Glacial and Interglacial Maxima**	#	Age	T1-MIS1	#	Age	T2 - MIS5	#	Age	T3 - MIS7	#	Age	T4 - MIS9*	#	Age	T5 - MIS11	ALL
SST Glacial (Summer-MAT)			*n.a.*			2.51			1.33			1.61			1.33	1.69
SST Glacial (Mg/Ca raw)	5	19.7-21.1 ka	1.59	2	137.3-138.5 ka	3.33	3	248.7-251.2 ka	2.05	2	355.7-359 ka	2.64	3	436.38-451.13 ka	2.34	2.39
SST Glacial (Mg/Ca corr)			-0.10			1.16			-0.65			0.61			-0.60	0.08
SST Interglacial (Summer-MAT)			*n.a.*			10.50			9.61			10.86			8.54	9.88
SST Interglacial (Mg/Ca raw)	3	6-11.9 ka	4.85	5	126.6-127 ka	6.91	2	240.8-242 ka	4.33	4	328.3-331.6 ka	5.17	4	404.35-408.35k a	6.58	5.57
SST Interglacial (Mg/Ca corr)			7.95			10.63			7.23			10.98			10.47	9.45
IG-G ΔSST (MAT-Summer)			*n.a.*			7.99			8.28			9.25			7.21	8.18
IG-G ΔSST (Mg/Ca raw)			3.26			3.58			2.28			2.53			4.23	3.18
IG-G ΔSST (Mg/Ca corr)			8.05			9.47			7.88			10.37			11.07	9.37

# number of samples averaged for mean values

** Mg/Ca values on NP for peak MIS9 are not available. To derive* ΔSST f*or MIS9 we used Mg/Ca values measured on N incompta, corrected using Morely et al. 2017.*

Supplementary Table 7. Materials and time intervals used to calculate ΔSST for the Holocene-LGM shown in Figure 3

LGM Core	Lat	Long	ΔSST	LGM SST	time interval [ka]	species LGM	Ref	Holo SST	time interval [ka]	species Holocene	ref	Holocene Core *(if different)*	Lat	Long
Rapid-15-4P	62.29	-17.13	-8.56	1.92	19.7-21.1	*N. pachy.*	*Thornalley et al 2010*	10.49	0-4	G. bulloides	*Thornalley et al 2009*			
RAPiD-10-1P	62.98	-17.59	-8.88	1.61	17.1-16.7*	*N. pachy.*	*Thornalley et al 2010*	10.49	0-4	G. bulloides	*Thornalley et al 2009*			
MD99-2227	57.48	-48.53	-6.26	-0.35	18-21.5	*N. pachy.*	*Winsor et al. 2012*	5.90	0-1.2	*N. pachy.*	*Moffa-Sanchez et al. 2014*	RAPiD-35-25B	57.5	-48.7
ODP980	55.48	-14.70	-7.80	5.89	18–21.5	*N. pachy.*	*Benway et al 2010*	13.70	0-2.3	G. bulloides	*Richter et al*			
GeoB18530	42.84	-49.23	-6.61	2.50	20-21**	*N. pachy.*	*Max et al. 2022*	9.11	0-4	*N. pachy.*	*Max et al. 2022*			
MD2011	66.97	7.64	-6.40	2.40	18–21.5	*N. pachy.*	*Meland et al. 2005*	8.80	0.2-0.8	*N. pachy.*	*Nyland et al. 2006*			
JM11-FI-19PC	62.83	-3.87	-4.57	1.18	18.3-21.5	*N. pachy.*	Ezat et al. 2017	5.74	0-4	N. pachy. [++]	Ezat et al. 2017			
HM 80-42	72.25	-9.19	-0.58	3.56	18–21.5	*N. pachy.*	*Meland et al. 2005*	4.14	core top	*N. pachy.*	*Meland et al. 2005*	HM80-43	72.25	-9.19
HM 94-25	75.6	1.32	-1.82	2.92	18–21.5	*N. pachy.*	*Meland et al. 2005*	4.74	core top	*N. pachy.*	*Meland et al. 2005*			
HM 94-30	74.38	-2	-1.90	0.78	18–21.5	*N. pachy.*	*Meland et al. 2005*	2.68	core top	*N. pachy.*	*Meland et al. 2005*	HM 94-34	-2.54	73.8
MD 2284	64.57	-0.72	-6.30	1.96	18–21.5	*N. pachy.*	*Meland et al. 2005*	8.26	10-11ka	*N. pachy.*	*Meland et al. 2005*			
HM 71-15	70	-17.43	-2.94	1.75	19-21.5	*N. pachy.*	*Meland et al. 2005*	4.70	core top	*N. pachy.*	*Meland et al. 2005*	M23351	70.36	-18.21
HM 52-43	64.25	0.73	-3.58	2.09	19-21.5	*N. pachy.*	*Meland et al. 2005*	5.67	core top	*N. pachy.*	*Meland et al. 2005*	HM16132	64.57	-0.72
HM 100-7	61.67	-4.72	-7.90	2.94	19-21.5	*N. pachy.*	*Meland et al. 2005*	10.84	core top	*N. pachy.*	*Meland et al. 2005*	HM133-40l	61.14	-2.15
HM 71-12	68.43	-13.87	0.31	2.97	19-21.5	*N. pachy.*	*Meland et al. 2005*	2.67	core top	*N. pachy.*	*Meland et al. 2005*			
PS 21842	69.45	-16.52	-0.40	2.95	19-21.5	*N. pachy.*	*Meland et al. 2005*	3.36	core top	*N. pachy.*	*Meland et al. 2005*	HM57-05	69.14	-13.12
HM 94-18	74.5	5.7	0.06	3.55	19-21.5	*N. pachy.*	*Meland et al. 2005*	3.49	core top	*N. pachy.*	*Meland et al. 2005*			
HM 94-13	71.63	-1.62	0.21	3.35	19-21.5	*N. pachy.*	*Meland et al. 2006*	3.15	core top	*N. pachy.*	*Meland et al. 2006*	HM94-12	71.32	-3.55
NEAP 8 K	59.47	-23.54	-5.82	5.08	19-21.5	*N. pachy.*	*Meland et al. 2006*	10.90	0-4	G. bulloides	*Barker and Elderfield, 2002*			
SO 82-4	59.1	-30.48	-7.84	3.49	19-21.5	*N. pachy.*	*Meland et al. 2006*	11.33	0-4	G. bulloides	*Farmer et al. 2008*	MD99-2155	57.43	−27.9

** end of record*

*** reduced interval to avoid subsurface warming signal preceeding H1*

[+] *added 10% to raw Mg/Ca before correction to account for reductive cleaning*

[++] *added 15% to raw Mg/Ca before correction to account for reductive cleaning and multiple leaches*

Supplementary Table 8. Hydrographic data, stable isotopes and Mg/Ca used for the reanalysis of Kozdon et al. [5] See also Fig. 1

Site	Long	Lat	Core depth	Hydrographic data CARINA [T, S, ALK, DIC, Silicate, Phosphate]				Hydrographic data Legrande [d18Osw]			Calc depth	T	S	d18O sw	Mg/Ca	CO3	d18Oc VPDB
			[m]	stations	date	Long	Lat	Stations	Long	Lat	[m]	[C]	[psu]	[‰]	[mmol/mol]	[mmol/kgSW]	[‰]
HM71-17	-13.02	70.00	1460	7; 9; 91	02/06/2002; 18/06/2002	-13.00; -11.82; -14.02	68.68; 69.59; 70,01	597; 598; 599	-12.5; -13.3; -14.5	69.5; 70.5	36.4	0.57	34.75	0.10	1.00	124.35	3.4
HM80-43	-9.19	72.25	2448	16; 489; 491	03/06/2002; 08/08/1998	-8.53, -11.00; -7.05	71.75; 73.00; 73.00	742; 744; 746	-7.5; -9.5; -11.5	71.5; 73.5	9.2	0.50	34.00	-0.04	0.98	127.24	3.5
23235-1	1.39	78.87	2500	31; 32; 33	07/06/2002	2.42; 2.54; 1.85	77.95; 78.37; 78.81	1251; 1252; 1253	0.5; 1.5; 2.5	78.5; 79.5	20.6	1.63	34.99	-0.33	0.90	138.67	3.03
HM94-30	-2.00	74.38	3599	502; 510; 33; 36	15&18/08/1998; 01&02/08/1996	-2.99; -2.04; -1.52; -3.01	73.99; 75.00	963; 964; 965	-1.5; -2.5; -3.5	74.5; 75.5	22.4	-0.12	34.59	0.11	0.92	140.16	3.67
HM94-12	-3.55	71.32	1816	18	04/06/2002	-7.12	72.52	746; 748; 750	-3.5; -5.5; -7.5	71.5; 73.5	7.0	0.88	34.64	0.09	0.80	142.95	3.23
HM97-948	7.64	66.97	1048	117, 118, 119	23/06/2002	6.29; 7.58; 8.83	66.00	405; 406; 407	6.5; 7.5; 8.5	66.5; 67.5	62.6	8.92	35.16	0.37	0.82	160.95	2.01
HM52-18	-14.14	62.27	1672	195; 197	07/10/2003	-17.84; -17.86	62.83	25; 27; 29	-14.5; 16.5; -18.5	61.5; 62.5	931.3	4.79	35.65	0.44	0.82	160.95	3.09
HM16132	-0.72	64.57	2798	110; 126; 217	22&25/06/2002; 12/10/2003	0.5898; -1.62; 1.79	66.00; 65.12; 63.90	255; 256; 258	-1.5; -0.5; 1.5	64.5; 65.5	66.7	6.74	35.07	0.39	0.80	153.05	2.58
HM57-11	-8.30	67.12	1617	100; 101; 102; 191	20/06/2002; 01/10/203	-9.64; -8.47; -7.98; -9.24	66.00; 68.67	389; 390; 391	-7.5; -8.5; -9.5	66.5; 68.5	35.1	4.12	34.66	0.25	0.80	155.38	3.19
HM57-20	1.67	62.65	750	217; 221; 223	12/10/2003; 13/10/2003	1.79; 3.41; 3.92	63.90; 63.19;62.96	116; 117; 118	1.5; 2.5; 350	62.5; 63.5	298.5	4.87	35.05	0.47	0.85	139.48	2.92
HM94-16	5.37	73.23	2356	158; 497; 500; 27	26/09/2003; 10&14/08/1998; 31/07/1996	6.98; 5.00; 4.94; 6.00	74.50; 73.00; 74.01; 74.00	900; 901; 902	4.5; 5.5; 6.5	73.5; 74.5	33.9	4.13	34.78	0.30	0.87	157.61	3.32
HM94-18	5.70	74.50	2469	158; 500; 512; 513	26/09/2003; 14-18-19/08/1998	6.98; 4.94; 4.00; 6.96	74.50; 74.01; 75.00; 75.02	971; 972; 973	4.5; 5.5; 6.5	74.5; 75.5	47.4	3.03	34.85	0.28	0.91	151.05	3.47
HM49-15	-0.36	66.34	3260	1009; 110; 111; 126	22/06/2002; 25/06/2002	-1.00; 0.59; 1.84; -1.62	66.00; 65.12	326; 327; 328	-1.5; -0.5; 0.5	65.5; 66.5	33.3	8.38	35.09	0.38	0.91	171.46	2.23
HM16142	2.60	63.25	1100	217; 221; 223	12&13/10/2003;	1.79; 3.41; 3.92	63.90; 63.19; 62.96	116; 117; 118	1.5; 2.5; 3.5	62.5; 63.5	63.9	9.73	35.19	0.40	0.93	175.33	1.83
HM52-39	-6.79	65.57	2305	102; 104; 105; 106	20&21/06/2002	-7.98; -6.63; -6.04; -5.28	66.00	320; 321; 322	-7.5; -6.5; -7.5	65.5; 66.5	22.2	5.99	34.83	0.32	0.97	162.92	2.73
23261-2	13.11	72.18	2224	498	12/08/1998	10.92	73.11	764; 765; 767	10.5; 11.5; 13.5	71.5; 73.5	37.4	6.86	35.04	0.32	0.97	167.87	2.55

Supplementary Table 8. Cont.

Site	Long	Lat	Core depth	Hydrographic data CARINA [T, S, ALK, DIC, Silicate, Phosphate]				Hydrographic data Legrande [d18Osw]			Calc depth	T	S	d18O sw	Mg/Ca	CO3	d18Oc VPDB
			[m]	stations	date	Long	Lat	Stations	Long	Lat	[m]	[C]	[psu]	[‰]	[mmol/mol]	[mmol/kgSW]	[‰]
23259-3	9.30	72.02	2518	498	12/08/1998	10.92	73.11	833; 834; 835	8.5; 9.5; 10.5	72.5; 73.5	34.4	7.20	35.03	0.34	1.01	171.20	2.5
HM16130	-2.42	65.10	3182	108; 126; 127; 128	21/06/2002; 25/06/2002	-2.67; -1.62; -2.36; -2.36; -3.06	66.01; 65.12; 64.72; 64.28	253; 254; 255	-3.5; -2.5; -1.5	64.5; 65.5	31.3	7.84	34.97	0.38	0.99	167.83	2.4
HM52-42	-2.80	66.34	3104	108; 109; 125	21-22-25/06/2002	-2.67; -1.00; -0.92	66.00; 66.01; 65.49	324; 325; 326	-3.5; -2.5; -1.5	65.5; 66.5	21.9	8.82	34.97	0.37	1.04	178.50	2.38
HM71-22	-3.61	69.34	1833	81; 83; 84; 190	16-17/06/2002; 01/10/2003	-0.60; -3.44; -4.93; -6.77	70.00; 69.24	606; 608; 610	-5.5; -3.5; -1.5	69.5; 70.5	43.4	6.94	35.09	0.29	1.04	168.53	2.44
HM57-16	-4.37	67.28	2816	104; 105; 106; 108	21/06/2002	-6.63; -6.04; -5.28; -2.67	66.00; 66.01	392; 393; 394	-6.5; -5.5; -4.5	66.5; 67.5	20.1	6.96	34.88	0.31	1.07	167.61	2.59
